# Supplementary material for: mRNA Isoforms and Variants in Health and Disease
Source: Int J Mol Sci. 2025 Sep 25;26(19):9356. doi: 10.3390/ijms26199356 (PMC12524470; doi:10.3390/ijms26199356)
Supplement: Supplementary file 1 [file ijms-26-09356-s001.zip › ijms-3848567-supplementary.pdf]

Table S1. Mechanisms of generating the mRNA isoforms

**Mechanism**	**Molecular Basis**	**Example Genes**	**Functional Outcome**	**References**
Alternative transcription start site (ATS)	Differential promoter usage at the 5′ end of the gene	NR5A1, KLF4	Controls tissue-specific expression	[39, 40, 102, 103]
Alternative splicing (ASP)	Exon skipping, intron retention, alternative exon usage	RBM20, MAPT, CFTR	Protein domain variation; pathogenic splice variants	[29, 34, 98, 108, 127, 140, 164]
Alternative polyadenylation (APA)	Usage of different poly(A) signals at 3′ end	HMGA2, SERPINA1	Alters 3′UTR length, mRNA stability, and translation	[46, 57, 116, 117]
Alternative transcription termination (ATT)	Premature transcription termination	MIA3	Isoforms with altered stability	[104, 105]

Table S2. The physiological roles of mRNA isoforms

Biological Stage	Genes	Key Regulatory Factors	Isoform Changes	Functional Outcomes	References
Early development and zygotic gene activation (ZGA)	*Pou5f1 (Oct4), Sall4, Dnmt1, F11r, Magi1*	ASP, RBPs (e.g., CSDE1), SF3B1, RBFOX2	Alternative splicing generates isoforms involved in pluripotency and DNA methylation. Coding isoform switches	Initiation of ZGA, early cell fate determination. Modulate cell-cell interactions during ZGA.	[25-29]
	CSDE1	Exon skipping during ASP	Maternal transcript clearance	Transition from maternal to zygotic control	[27,28]
Organogenesis	RBM20, NOVA2, SRF, NR5A1, RUNX, BAD	TFs (e.g., SRF, NR5A1), ASP regulators	Isoforms affect cardiac and neural development. NR5A1 isoforms drive gonadal/adrenal development	Organ-specific morphogenesis; disorders of sex determination, if dysregulated	[34-40]
	HMGA2	APA	Shorter 3′ UTR isoforms with higher translational efficiency	Supports rapid protein production during development	[46, 47]
Normal physiology	CACNA1C	ASP, RBPs (SRSF1, hnRNPs)	Tissue-specific isoforms in cardiac vs. smooth muscle	Maintains proper cardiac and vascular function	[58, 59]
	Dscam (Drosophila)	Extreme ASP	More than 38,000 isoforms	Neural circuit specificity and connectivity	[61, 62]
	TBX20	APA, tissue-specific regulation	Isoform expressions vary across tissues	Regulates cardiac and reproductive system function	[63]
	HSP70	m6A modification	Cap-independent translation via 5′ UTR m6A	Rapid stress response (e.g., heat shock)	[64-67]
Aging	*Cdkn1a* (P21), ESRRG, TET2	Histone modifiers (H3K36me), Pol II, RBPs (HuR, TIA-1)	Isoform shifts (e.g., Cdkn1a variant 2 increases); disrupted splicing in ESRRG, TET2	Promotes senescence, affects muscle development, and reduces repair capacity	[71-83]

Table S3. Representative splicing mechanisms, key factors, and disease-specific variants

Disease Context	Splicing Mechanism	Key Splicing Factors	Resulting Variant Outcome	References
Neurological diseases (AD, developmental disorders)	Aberrant ASP of MAPT and APP pre-mRNAs	RBPs / SPFs dysfunction (e.g., SF3B1, RBFOX2)	Tau variants (tangles in AD); altered APP isoform ratios linked to plaque formation	[98, 99, 101]
Cardiovascular diseases (heart failure, hypertrophy)	ASP of sarcomeric genes TTN, TNNT2, MYH7	RBM20	Altered sarcomeric isoforms impair myocardial stiffness, contraction, and hypertrophy.	[108, 109, 110]
Asthma (respiratory disease)	ASP of ADAM33 pre-mRNA	RBP / SPFs driving exon loss	Isoforms lacking the catalytic domain of ADAM33 impair airway remodeling	[112]
COPD	Exon skipping, intron retention (ASP)	Impaired splicing in severe COPD	Aberrant isoforms influencing inflammation and remodeling	[115, 116, 117]
Genitourinary diseases	ASP in the kidney, ovary, and testis; compartment-specific splicing	ESRP1, ESRP2, RBFOX2	Transcript variants are found in kidney. FGFR2 and ARHGEF10L variants are linked to Wilms tumor. Isoforms in the ovary/testis regulate differentiation, hormone response, spermatogenesis, and folliculogenesis.	[125-130]
Musculoskeletal diseases (DMD, SMA, ALS, congenital myopathy)	ATS and ASP of Dystrophin, SMN, FXR1, ALS genes	hnRNPs, splicing machinery	Isoforms lacking normal dystrophin; Reduced SMN proteins; FXR1 isoforms in congenital multi-minicore myopathy; ALS-related RBP misregulation	[135-143]
Metabolic diseases (Diabetes)	Mis-regulated ASP of insulin regulatory genes	RBPs (e.g. HuR)	Pathogenic isoforms impair β-cell maturation and insulin signaling; aberrant isoforms are implicated in complications.	[144-150]
Genetic diseases (Down, CF, thalassemia, Tay-Sachs)	ASP, mutations at splice sites, APA disruption	Dysregulation of splicing regulators (e.g., DYRK1A in Down); general splicing machinery	Down syndrome: transcriptome-wide shifts, DYRK1A-driven global splicing aberrations, altered APP/BIN1 isoforms; CF: CFTR splicing or polyA mutations produce nonfunctional variants; Thalassemia: β-globin mis-splicing, cryptic splice sites, unstable mRNAs; Tay-Sachs: HEXA splicing disruption, loss of function	[157-168]
Autoimmune disease (SLE, RA, MS)	ASP defects of immune-regulatory transcripts	Dysregulation of IRF5, CTLA4, CD44, TNFRSF1B SPFs	Pathogenic isoforms enhancing autoimmunity and inflammation	[172, 173, 174, 179]
Infectious diseases (HIV, MTB, EBV, Influenza)	Viral manipulation of host ASP and ATS	Host RBPs / splicing machinery hijacked	HIV - CCNT1 and CCR5 variant;<br>MTB- RAB8B and IL-12Rβ variant;<br>EBV- MPPE1 variant;<br>Influenza- Cap-snatching;<br>HBV- SP1 variant;<br>Listeria- CIRBP isoforms.	[195-199, 202, 203, 210, 211, 212]

Cancer (lung, uterine, glioblastoma, liver, breast, prostate, skin, bladder, melanoma)	Aberrant ASP in oncogenes/tumor suppressors	RBM20 (cardiac tumors), SPFs are generally dysregulated	Oncogenic activation: RAF1, FOS, BCL2L1, BCL-XL, EGFRvIII, CDK5, SRP19, CDKN1A, and SMS. Tumor suppressor inactivation: GLTSCR2, EMP3, TMPRSS2-ERG fusions	[224-245]

Table S4. Representative splicing isoforms, genes, and functions

Gene	Isoform / Splicing Event	Biological or Disease Function	References
RBFOX2	Various isoforms (regulated by ASP)	Splicing factor critical for development; its dysregulation leads to cancer.	[29]
MAPT	Tau isoforms (ASP variants)	Different tau isoforms aggregate into neurofibrillary tangles in Alzheimer's disease.	[98, 99]
RBM20	TTN, MYH7, TNNT2 variants (cardiac genes)	Regulates cardiac muscle development and disease; mis-splicing leads to heart failure	[34, 110]
CYP27B1	Vitamin D receptor isoforms (ASP)	Defective splicing causes vitamin D-dependent rickets	[134]
DMD	Various dystrophin isoforms	Splicing defects generate nonfunctional forms in Duchenne Muscular Dystrophy	[137, 138]
SMN1/2	SMN1/2 variable exon7 inclusion	Splicing determines functional protein level; critical for spinal muscular atrophy	[140, 141]
CFTR	Mis-spliced CFTR isoforms	Aberrant splicing produces nonfunctional CFTR in cystic fibrosis	[164, 165]
IRF5	Proinflammatory splice isoforms	Overexpressed in SLE and drives autoimmune responses	[172, 173]
OAS1	p42/p44/p48 isoforms (vs p46)	Altered isoform expression affects autoimmunity in Sjögren's syndrome	[175]
FOS	Cancer-specific FOS transcript variants	Splicing promotes cell proliferation in uterine cancer	[225]
BCL2L1	BCL-XL (anti-apoptotic) and BCL-XS	ASP produces anti-apoptotic BCL-XL; it is upregulated in various cancers	[226]
EGFR	EGFRvIII	Tumor-specific variant drives glioblastoma growth; absent in normal tissues	[231, 232, 233]
ACY1	Brain cancer-specific ACY1 isoforms	Mis-splicing promotes cell cycle dysregulation in tumors	[234]
CDKN1A	Cytoplasmic p21 (spliced variant)	Mis-localized isoform impairs tumor suppression in lung cancer	[237]
TMPRSS2-ERG	Oncogenic fusion transcript	Splicing-mediated fusion drives prostate cancer via ERG activation	[241, 242, 243]
